# Transmembrane and Ubiquitin-Like Domain-Containing 1 Promotes Glioma Growth and Indicates Unfavorable Prognosis

**DOI:** 10.1155/2023/3318171

**Published:** 2023-12-19

**Authors:** Yinggang Liu, Changcheng Cai, Ke Wu, Libo Hu

**Affiliations:** ^1^Department of Neurosurgery, Suining Central Hospital, Suining 629000, Sichuan, China; ^2^Department of Neurosurgery, Xichang People's Hospital, Xichang 615000, Sichuan, China

## Abstract

**Background:**

Ubiquitin-related proteins have garnered increasing attention for their roles in tumorigenesis. Transmembrane and ubiquitin-like domain-containing 1 (TMUB1) is a recently discovered protein in the ubiquitin-like domain family, yet its involvement in glioma remains poorly understood. This study is aimed at investigating the functional significance and clinical relevance of TMUB1 in glioma.

**Methods:**

We conducted a comprehensive analysis using two cohorts: a retrospective glioma cohort from our hospital and The Cancer Genome Atlas (TCGA) cohort. The mRNA levels of TMUB1 were assessed through reverse transcription-quantitative polymerase chain reaction (RT-qPCR). Clinical associations of TMUB1 in these cohorts were evaluated using correlation tests, chi-square tests, and survival analyses. Additionally, we performed TMUB1 knockdown in U87 and LN-229 human glioma cell lines, and cellular growth was assessed through the 3-(4,5-dimethylthiazol-2-yl)-2,5-diphenyl-2H-tetrazolium bromide (MTT) assay.

**Results:**

Our results revealed that TMUB1 expression was elevated in glioma tissues compared to normal brain tissues. Notably, lower TMUB1 expression correlated with favorable characteristics such as lower World Health Organization (WHO) grade and 1p/19q codeletion. Moreover, patients with higher TMUB1 levels in glioma tissues exhibited worse prognosis in both TCGA cohort and our retrospective cohort, underscoring its prognostic significance in gliomas. Cellular experiments demonstrated that TMUB1 silencing suppressed the growth of glioma cells.

**Conclusions:**

TMUB1 emerges as a novel and clinically relevant prognostic biomarker for gliomas. Targeting TMUB1 holds promise as a potential strategy for glioma treatment. This study contributes valuable insights into the multifaceted role of TMUB1 in glioma pathogenesis and its potential as a diagnostic and therapeutic target.

## 1. Introduction

Gliomas represent a diverse group of primary brain tumors that arise from glial cells, which provide crucial support and protection to neurons in the central nervous system [1]. These tumors are associated with a spectrum of neurological symptoms, depending on their location within the brain. Gliomas are known for their infiltrative nature, often making complete surgical removal challenging [2]. They encompass various subtypes, with glioblastoma (GBM) being one of the most aggressive and common forms. GBM, a highly malignant solid tumor that originates from astrocytes within the spinal cord and brain tissues, presents a formidable challenge in the field of oncology. GBM is typically diagnosed in older individuals, with the median age of diagnosis above 50 years old [3]. Nevertheless, it is essential to note that GBM can also manifest in children, underscoring its potential impact across various age groups. The prognosis and treatment options for glioma patients vary widely based on factors such as tumor grade, location, and molecular characteristics. For example, studies have revealed that only 5% achieves the milestone of a 5-year survival [4]. Consequently, the overwhelming majority of GBM patients face a dire prognosis, with the disease often proving fatal shortly after diagnosis. Therefore, there is an urgent need for comprehensive research to improve our understanding and management of glioma [5].

TMUB1 plays a pivotal role in endoplasmic reticulum- (ER-) associated protein degradation (ERAD), a cellular quality control mechanism responsible for identifying and targeting misfolded or unwanted proteins for degradation [6]. As part of the ERAD machinery, TMUB1 aids in the recognition and ubiquitination of aberrant proteins, facilitating their disposal by the proteasome [7]. Moreover, TMUB1 possesses a transmembrane domain that allows it to anchor to the ER membrane, where it interacts with various ER-resident proteins involved in ERAD [8]. This interaction is essential for maintaining ER homeostasis and ensuring the proper folding and processing of proteins within the ER, thereby preventing the accumulation of unfolded or misfolded proteins that could be detrimental to the cell [9, 10].

In recent years, research has highlighted the potential involvement of TMUB1 in several diseases, such as the inflammation-related recurrent spontaneous abortion [11]. Notably, studies have shown a connection between TMUB1 and colorectal cancer, which shows oncogenic potentials [12]. Nevertheless, the role of TMUB1 in hepatocellular cancer seems to be antioncogenic [13, 14]. Although the tumor-related role of TMUB1 in other malignancies was slightly mentioned in lung cancer [15] and pancreatic cancer [16], direct evidence was lacking, especially whether TMUB1 contributes to glioma development and progression is still unclear. However, emerging evidence suggests that it may play a role in the regulation of key cellular processes, such as apoptosis, cell cycle control, and DNA repair, which are critical for glioma pathogenesis. Understanding the role of TMUB1 in malignancies is crucial, as it may provide insights into the molecular mechanisms driving the disease and potentially open new avenues for therapeutic interventions.

In this paper, we delve into the pivotal role of TMUB1 in glioma pathogenesis. Through a comprehensive analysis of clinical data, functional experiments, and immune infiltration assessments, we shed light on the multifaceted involvement of TMUB1 in glioma progression and patient outcomes. Our investigation revealed the clinical relevance of TMUB1 as a potential diagnostic and prognostic marker in glioma, emphasizing its critical role as a prognostic indicator. Functional experiments provided mechanistic insights into TMUB1's oncogenic role, and our analysis unveiled correlations between TMUB1 expression and immune cell infiltration in the glioma microenvironment. Furthermore, our univariate analysis identified WHO grade and TMUB1 expression as significant predictors of overall survival, highlighting the potential of TMUB1 as a promising biomarker and therapeutic target in the challenging landscape of glioma, with implications for risk stratification and personalized treatment approaches.

## 2. Methods

### 2.1. Data Collection

Gene expression profiling data for glioma samples and normal brain samples were obtained from The Cancer Genome Atlas (TCGA) database (https://tcga-data.nci.nih.gov/tcga/). Complete clinical information, including survival data, was also acquired [17].

### 2.2. Tissue Specimen Collection

A total of 109 glioma tissue specimens were collected from our hospital. The pathological diagnosis of glioma was confirmed for all patients. This study was conducted following approval from the Ethics Committee of Suining Central Hospital, and all patients provided informed consent. The inclusion criteria encompass glioma patients from Suining Central Hospital with confirmed pathological diagnosis, complete clinical information, and available TMUB1 mRNA levels assessed through RT-qPCR. In the retrospective cohort, inclusion criteria consider tumor characteristics such as size, WHO grade, 1p/19q codeletion status, and IDH1/2 status. Exclusion criteria involve incomplete clinical data, insufficient or unreliable TMUB1 data, nonglioma diagnoses, unavailability of survival data, and the inclusion of other cancer types.

### 2.3. RNA Extraction and RT-qPCR

Total RNA extraction was performed using the RNAstorm™ RNA Isolation Kit (Biotium) according to the kit's instructions. RNA integrity and concentration were assessed using a 2100 Bioanalyzer. All RNA samples exhibited a concentration exceeding 2,000 *μ*L per 1 *μ*L of water. For cDNA preparation, 4 *μ*L of RNA samples was used as templates for reverse transcription using the SSRT III system (Thermo Fisher). Quantitative PCR (qPCR) was carried out to measure TMUB1 expression, with GAPDH as the internal control. Relative gene expression levels were calculated using the 2-delta delta Ct method. Primer sequences used were as follows: TMUB1 (F: 5′-CCTCAATGATTCAGAGCAGGTGG-3′; R: 5′-AGATGAGTCGCACCTGCTGTTC-3′, OriGene, CAT#: HP215583) and GAPDH (F: 5′-GTCTCCTCTGACTTCAACAGCG-3′; R: 5′-ACCACCCTGTTGCTGTAGCCAA-3′, OriGene, CAT#: HP205798).

### 2.4. Cell Culture

Human glioma cells (LN-229, U87) were obtained from the Shanghai Bank of Cell Culture (Chinese Academy of Sciences, China). Cells were cultured in DMEM supplemented with 10% fetal bovine serum (FBS, Gibco, USA) at 37°C in a humidified atmosphere containing 5% CO_2_ [18].

### 2.5. TMUB1 Knockdown

Lentiviruses containing short hairpin RNAs (shRNAs) targeting TMUB1 were obtained from GenePharma Co., Ltd. (Shanghai, China) with the sequence 5′-GACACCATTGGCTCCTTGAAA-3′ [13]. A scrambled sequence (5′-AACTTAGCCAGCTAGGGC-3′) was employed as a negative control. The shRNAs were cloned into the pGIPZ lentivector (GenePharma Co., Ltd.). Lentiviruses were generated following the manufacturer's instructions using a lentivector kit (Lenti-Pac™; iGene Biotechnology Co., Ltd.). Lentiviruses were used to infect LN-229 and U87 cells, and transfected cells were selected using puromycin.

### 2.6. Proliferation Assay

Cell proliferation was assessed using the 3-(4,5-dimethylthiazol-2-yl)-2,5-diphenyl tetrazolium bromide (MTT) assay. Briefly, cells were seeded in 96-well plates at a density of 5,000 cells per well and cultured under standard conditions for specified time points (8 h, 24 h, 48 h, 72 h, and 96 h). At each time point, triplicate wells were treated with 150 *μ*L of MTT solution and incubated for an additional 15 minutes. Following incubation, the MTT formazan was fully dissolved by gentle shaking and pipetting. The absorbance was measured at 570 nm using a spectrophotometer.

### 2.7. Statistical Analysis

Overall survival time was defined as the period from the time of diagnosis until the last date of follow-up or the date of death. Statistical analysis was performed using GraphPad Prism 5.0 (GraphPad Prism, CA, USA). Student's *t*-test was used to determine the statistical significance between two groups. Data presented are expressed as means ± standard deviation (SD) from a minimum of three independent experiments. A *P* value of ≤ 0.05 was considered statistically significant.

## 3. Results

### 3.1. Differential mRNA Levels of TMUB1 in Glioma Subtypes

**Figure 1:**
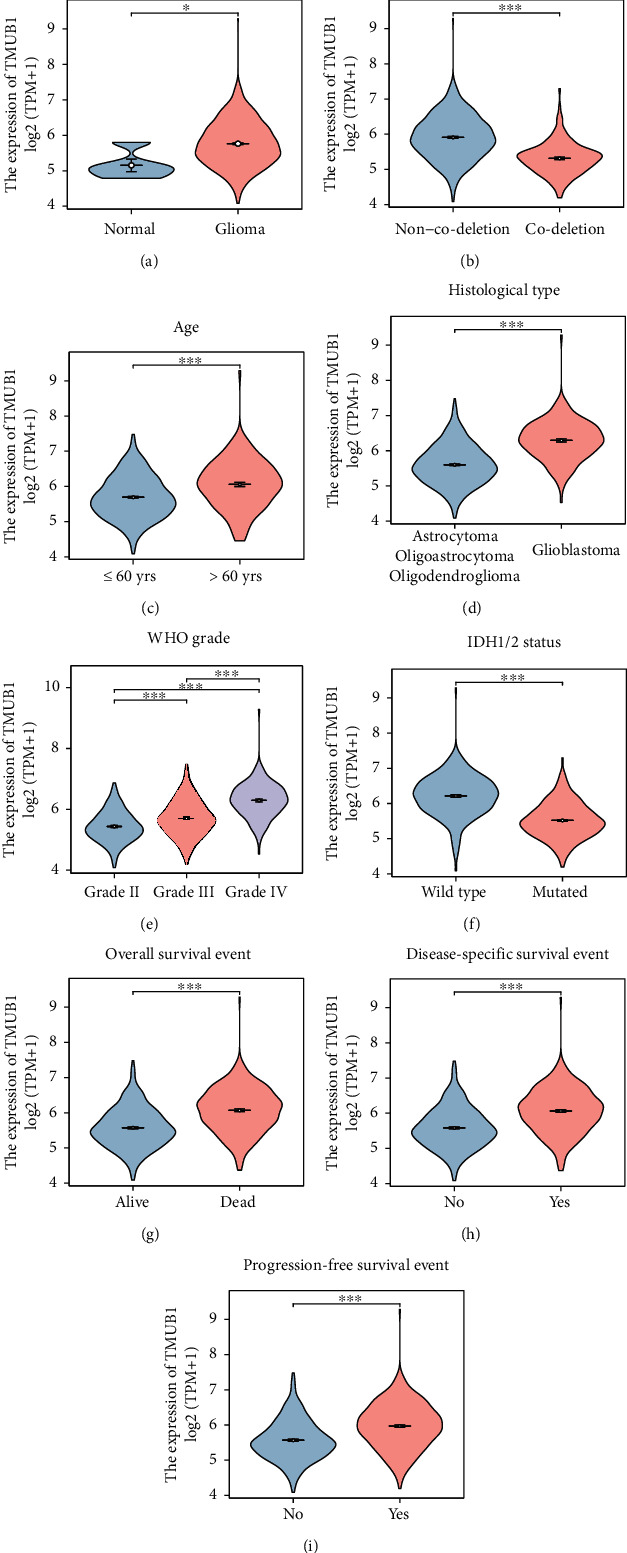
TMUB1 mRNA levels in glioma subtypes. (a) Comparative analysis illustrates a significant upregulation of TMUB1 mRNA levels in glioma tissues compared to normal brain tissues, indicating its potential involvement in glioma pathogenesis. (b) TMUB1 expression is markedly higher in glioma tissues lacking 1p/19q codeletion than in those with the codeletion, suggesting a potential association between TMUB1 levels and molecular subtypes of glioma. (c) Glioma patients aged over 60 exhibit a notable increase in TMUB1 expression in their tumor tissues, highlighting a potential age-related impact on TMUB1 expression. (d) TMUB1 demonstrates significantly higher expression in glioblastoma tissues compared to astrocytoma, oligoastrocytoma, and oligodendroglioma, indicating its potential relevance in the aggressiveness of glioma. (e) TMUB1 levels are significantly elevated in gliomas with higher World Health Organization (WHO) grades, suggesting its potential as a biomarker for glioma grading. (f) Glioma tissues with wild-type IDH1/2 status exhibit considerably higher TMUB1 expression compared to those with mutated IDH1/2, revealing a potential genetic association. (g) TMUB1 expression is notably higher in glioma patients who experienced mortality during the follow-up period, indicating its potential prognostic value. (h) Similarly, higher TMUB1 levels are observed in glioma patients who succumbed to disease-specific death, reinforcing its potential role as a prognostic indicator. (i) Patients with disease progression exhibit significantly increased TMUB1 expression in their glioma tissues, suggesting its involvement in glioma advancement. Data was statistically analyzed using the Wilcoxon rank sum test. ^∗^*P* < 0.05; ^∗∗∗^*P* < 0.001.

To investigate the mRNA levels of TMUB1 in glioma, we analyzed TCGA cohort of glioma tissues and compared them to normal brain tissues (Figure 1(a)). Our results revealed a significant upregulation of TMUB1 expression in glioma tissues, highlighting its potential role in glioma development. Subsequently, we examined TMUB1 expression in glioma tissues with and without 1p/19q codeletion (Figure 1(b)). Notably, TMUB1 expression was significantly higher in glioma tissues without 1p/19q codeletion, suggesting a potential association between TMUB1 levels and molecular subtypes of glioma. Age is an important factor in glioma prognosis, and in Figure 1(c), we observed a significant increase in TMUB1 expression in glioma tissues from patients older than 60 years, indicating a potential age-related impact on TMUB1 expression. We also compared TMUB1 expression in different glioma subtypes, including glioblastoma, astrocytoma, oligoastrocytoma, and oligodendroglioma (Figure 1(d)). Our analysis revealed significantly higher TMUB1 levels in glioblastoma compared to other subtypes, suggesting its potential relevance in the aggressiveness of glioma. The World Health Organization (WHO) grade is an important parameter for glioma classification.  Figure 1(e) demonstrates that TMUB1 expression significantly increased with higher WHO grades, further emphasizing its potential as a biomarker for glioma grading. Investigating the genetic aspect of gliomas, our data also indicates that TMUB1 expression was significantly higher in glioma tissues with wild-type IDH1/2 status compared to those with mutated IDH1/2 (Figure 1(f)). Moreover, we examined the association between TMUB1 levels and patient outcomes. Glioma patients who did not survive during the follow-up period exhibited significantly higher TMUB1 expression, highlighting a potential prognostic value (Figure 1(g)). Consistently, prognosis data illustrates that glioma patients who experienced disease-specific death had higher TMUB1 levels, further underscoring its potential role in predicting disease-specific outcomes (Figure 1(h)). Finally, we assessed TMUB1 expression in relation to disease progression (Figure 1(i)). Our data revealed that TMUB1 levels were significantly higher in glioma tissues from patients who experienced disease progression, suggesting its involvement in glioma advancement. These findings collectively underscore the multifaceted role of TMUB1 in glioma pathogenesis and its potential as a diagnostic and prognostic marker in TCGA cohort.

### 3.2. Correlations between TMUB1 and Patients' Characteristics in Our Retrospective Cohort

Furthermore, we conducted an analysis of the correlations between clinical characteristics and TMUB1 mRNA levels in the cohort of glioma patients from our hospital (Suining Central Hospital, SCH), aiming to further elucidate the potential associations between these factors and TMUB1 expression (Table 1).

Among the 109 glioma patients, 53 had low TMUB1 expression, and 56 had high TMUB1 expression according to the median cut-off value of mRNA level. There was no statistically significant association between gender and TMUB1 expression (*P* = 0.820), indicating that the distribution of TMUB1 expression levels was relatively balanced between female and male patients. Next, we assessed the influence of age on TMUB1 expression, which revealed no significant difference in TMUB1 expression levels based on age (*P* = 0.630) in our cohort, which is different with TCGA cohort.

Interestingly, the analysis of tumor size yielded significant results (*P* = 0.005). Patients with tumors larger than 5.0 cm exhibited a higher prevalence of high TMUB1 expression compared to those with smaller tumors, suggesting a positive correlation between larger tumor size and increased TMUB1 expression. Furthermore, we explored the relationship between TMUB1 expression and the WHO grade of glioma. The results demonstrated a significant association (*P* = 0.001) between higher WHO grades (grade III and grade IV) and increased TMUB1 expression, indicating that more advanced glioma grades were associated with elevated TMUB1 mRNA levels. In contrast, the Karnofsky score, a measure of performance status, did not show any significant correlation with TMUB1 expression (*P* = 0.688). Lastly, we examined the impact of different surgery patterns on TMUB1 expression. Gross total resection (GTR), subtotal resection (STR), or partial resection (PR/biopsy) was conducted in our cohort. The analysis revealed no significant association between these surgery patterns and TMUB1 expression (*P* = 0.366).

### 3.3. Prognostic Role of TMUB1 in Glioma Cohorts

**Figure 2:**
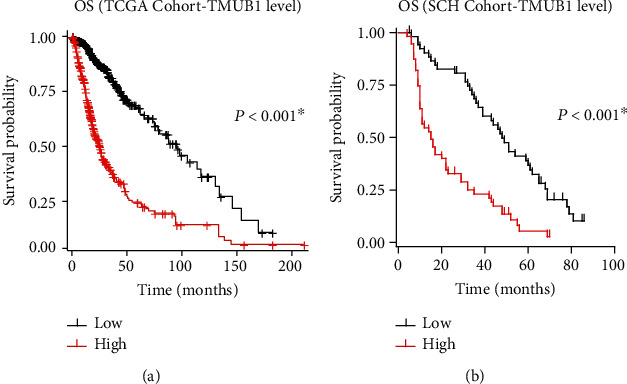
Overall survival analysis based on TMUB1 expression. (a) The Kaplan-Meier overall survival curve derived from TCGA-GBMLGG dataset, stratified by low and high TMUB1 mRNA levels. The curve underscores the inferior survival of glioma patients with elevated TMUB1 expression (^∗^*P* < 0.05). (b) The Kaplan-Meier overall survival curve for the Suining Central Hospital (SCH) cohort, categorized by low and high TMUB1 mRNA levels. The curve illustrates poorer survival outcomes among patients with increased TMUB1 expression. Statistical significance was determined using the log-rank test. ^∗^*P* < 0.05.

Considering that both TCGA cohort and our SCH cohort indicate that tumor size and WHO grade are significantly associated with TMUB1 expression, we next conducted survival analyses to further illustrate its clinical relevance. The Kaplan-Meier overall survival curve demonstrates a significant association between TMUB1 mRNA levels and patient survival in TCGA-GBMLGG dataset (Figure 2(a), *P* < 0.001). Patients with high TMUB1 expression exhibited significantly worse overall survival compared to those with low TMUB1 expression, as indicated by the log-rank test. These findings suggest that elevated TMUB1 expression is an adverse prognostic factor for glioma patients in TCGA dataset.

To validate our observations, we extended our analysis to an independent cohort from Suining Central Hospital (SCH). The Kaplan-Meier overall survival curve for this cohort was also stratified based on low or high TMUB1 mRNA levels. Consistent with TCGA results, patients with high TMUB1 expression in the SCH cohort also experienced significantly poorer overall survival (Figure 2(b), *P* < 0.001). In detail, patients with low TMUB1 expression exhibited a substantially longer median OS of 48.0 months and a 3-year OS rate of 66.4%. In contrast, patients with high TMUB1 expression had a markedly shorter median OS of 15.0 months and a 3-year OS rate of 23.1%. Collectively, the results from both datasets emphasize the clinical relevance of TMUB1 as a prognostic marker in glioma. High TMUB1 expression is consistently linked to worse overall survival, supporting its potential utility in risk stratification and treatment decision-making for glioma patients.

**Figure 3:**
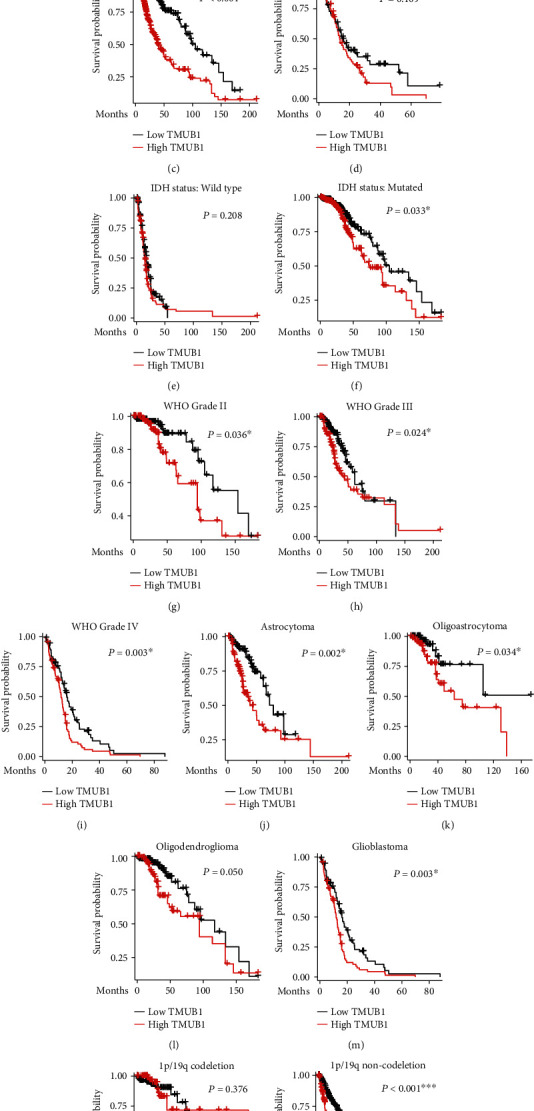
Prognostic significance of TMUB1 level in glioma patients of different subgroups. The Kaplan-Meier overall survival curves were constructed to assess overall survival in TCGA cohort with different characteristics, including females (a), males (b), younger patients (c), elder patients (d), IDH wild type (e), IDH mutated (f), WHO grade II (g), WHO grade III (h), WHO grade IV (i), astrocytoma (j), oligoastrocytoma (k), oligodendroglioma (l), glioblastoma (m), 1p/19q codeletion (n), and 1p/19q noncodeletion (o). Statistical significance was determined using the log-rank test. ^∗^*P* < 0.05.

Moreover, we conducted more detailed analyses of TMUB1's prognosis significance in different subgroups, which provided a more detailed vision of survival data. For example, the prognostic significance of TMUB1 level was tested in patients with different genders (Figures 3(a) and 3(b)), ages (Figures 3(c) and 3(d)), IDH status (Figures 3(e) and 3(f)), WHO grades (Figures 3(g)–3(i)), histological types (Figures 3(j)–3(m)), and 1p/19q codeletion or not (Figures 3(n) and 3(o)). In summary, high TMUB1 indicates significantly worse survival in all subgroups except patients elder than 60 years old, wild-type IDH status, or 1p/19q codeletion.

**Figure 4:**
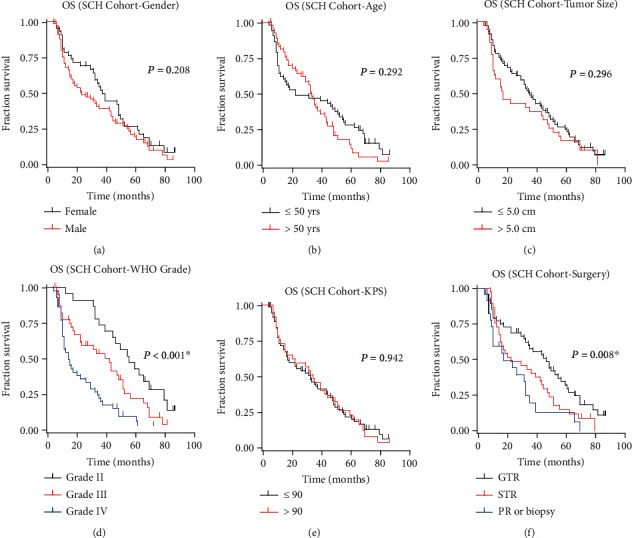
Overall survival analyses in the Suining Central Hospital cohort based on various variables. The Kaplan-Meier overall survival curves were constructed to assess overall survival in the Suining Central Hospital (SCH) cohort, stratified by different variables, including patients' gender (a), age (b), tumor size (c), WHO grade (d), Karnofsky Performance Status (KPS) score (e), and surgical pattern (f). Statistical significance was determined using the log-rank test. ^∗^*P* < 0.05.

Besides TMUB1, the prognostic significance of other variables in our cohort was also evaluated to provide a comprehensive understanding of the prognostic significance of various factors in glioma patients (Table 2). Accordingly, there was no statistically significant difference in OS between female and male patients (Figure 4(a), *P* = 0.208). The median OS for females was 37.0 months, with a 3-year OS rate of 51.8%, while for males, the median OS was 23.0 months, with a 3-year OS rate of 39.1%. The analysis of age and OS demonstrated no significant association (Figure 4(b), *P* = 0.292). Patients aged 50 or younger had a median OS of 22.0 months and a 3-year OS rate of 47.2%, while those older than 50 had a median OS of 33.0 months and a 3-year OS rate of 40.3%. Tumor size also did not significantly impact OS (Figure 4(c), *P* = 0.296). Patients with tumors smaller than or equal to 5.0 cm had a median OS of 35.0 months and a 3-year OS rate of 47.4%, while those with tumors larger than 5.0 cm had a median OS of 17.0 months and a 3-year OS rate of 37.2%. In contrast, the WHO grade of gliomas had a significant impact on OS (Figure 4(d), *P* < 0.001). Grade II gliomas had a notably longer median OS of 65.0 months, with a 3-year OS rate of 73.9%. Grade III gliomas had a median OS of 42.0 months and a 3-year OS rate of 53.8%, while grade IV gliomas had the shortest median OS of 15.0 months, with a 3-year OS rate of 20.5%. The Karnofsky score, reflecting patient performance status, did not significantly affect OS (Figure 4(e), *P* = 0.942). Patients with a Karnofsky score of 90 or lower had a median OS of 32.0 months, with a 3-year OS rate of 43.2%, while those with a Karnofsky score greater than 90 had a median OS of 34.0 months, with a 3-year OS rate of 45.9%.

Finally, the type of surgical resection exhibited a significant impact on OS (Figure 4(f), *P* = 0.008). Patients who underwent gross total resection (GTR) had a longer median OS of 46.0 months, with a 3-year OS rate of 57.7%. Those who underwent subtotal resection (STR) had a median OS of 22.0 months and a 3-year OS rate of 40.5%, while patients with partial resection (PR/biopsy) had the shortest median OS of 17.0 months and a 3-year OS rate of 19.8%. In summary, WHO grade, surgery type, and TMUB1 mRNA level are identified as significant predictors of OS, emphasizing their clinical relevance in determining patient outcomes.

Furthermore, we conducted multivariate Cox regression analysis to help identify independent prognostic factors (Table 3). Accordingly, the WHO grade, surgery type, and TMUB1 mRNA level were evaluated as independent variables. Notably, grade IV gliomas exhibited a hazard ratio (HR) of 2.995 (95% CI: 1.609-5.575, *P* = 0.001), signifying a significantly higher risk of adverse outcomes compared to grade II gliomas. Similarly, patients undergoing partial resection (PR/biopsy) demonstrated a pronounced HR of 2.575 (95% CI: 1.451-4.568, *P* = 0.001), indicating a substantial increase in the risk of unfavorable prognosis compared to those who underwent gross total resection (GTR), the reference category. Moreover, high TMUB1 mRNA levels were associated with a noteworthy HR of 2.389 (95% CI: 1.504-3.796, *P* < 0.001), underlining the robust prognostic significance of elevated TMUB1 expression in gliomas. These findings emphasize the potential utility of TMUB1 as a prognostic biomarker with implications for risk stratification and treatment decision-making.

### 3.4. Knockdown of TMUB1 Inhibits Glioma Cell Growth

**Figure 5:**
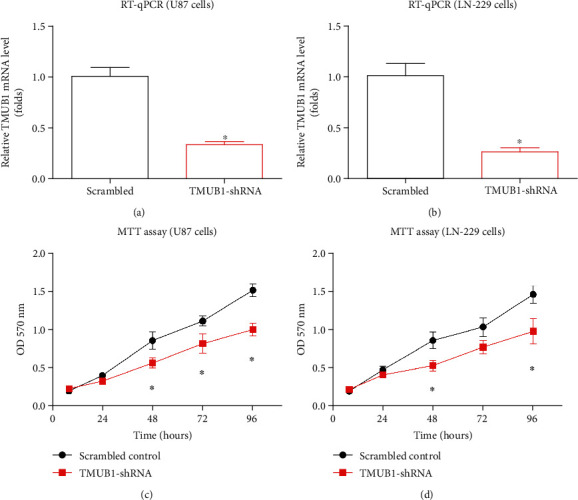
Inhibition of glioma cell growth through TMUB1 knockdown. (a, b) RT-qPCR experiments were carried out to validate the effectiveness of TMUB1-shRNA in knocking down TMUB1, with a comparison to the scrambled-shRNA group, in U87 and LN-229 glioma cells. ^∗^*P* < 0.05. (c, d) Cellular growth capacity of U87 and LN-229 cells was assessed through MTT experiments, demonstrating a significant suppression of cell growth upon TMUB1 silencing.

To investigate the functional impact of TMUB1 on glioma cell growth, we employed a knockdown approach and assessed its effects on cellular proliferation. We initially conducted RT-qPCR experiments to validate the knockdown efficiency of TMUB1 using TMUB1-shRNA, comparing it to the control group treated with scrambled-shRNA. This experiment was carried out in U87 and LN-229 glioma cell lines. The RT-qPCR results confirmed a substantial reduction in TMUB1 mRNA levels in the TMUB1-shRNA-treated cells compared to the control group (Figures 5(a) and 5(b)). This reduction validated the successful knockdown of TMUB1 in both U87 and LN-229 cells.

Following the successful knockdown of TMUB1, we proceeded to evaluate the impact of TMUB1 silencing on glioma cell growth using MTT experiments. The MTT assays clearly demonstrated that silencing TMUB1 led to a significant reduction in cellular growth capacity in both U87 and LN-229 cells (Figures 5(c) and 5(d)). These results indicate that TMUB1 plays a crucial role in promoting glioma cell proliferation. The knockdown of TMUB1 not only confirmed its influence on glioma cell growth but also provides valuable insights into the potential therapeutic strategies targeting TMUB1 for glioma treatment. These findings contribute to our understanding of the functional role of TMUB1 in glioma pathogenesis and highlight its significance as a potential therapeutic target.

## 4. Discussions

Glioma, a devastating form of brain cancer, poses substantial clinical challenges. In this study, we explored the multifaceted role of transmembrane and ubiquitin-like domain-containing 1 (TMUB1) in glioma, shedding light on its potential as a diagnostic biomarker, a prognostic indicator, and even a therapeutic target. Our findings, derived from a comprehensive analysis of clinical data, functional experiments, and immune infiltration assessments, offer valuable insights into the complex landscape of glioma.

The significant upregulation of TMUB1 in glioma tissues compared to normal brain tissues highlights its potential as a diagnostic marker. Elevated TMUB1 expression is linked to unfavorable clinical features such as higher WHO grades, advanced age, larger tumor size, and wild-type IDH1/2 status. This aligns with prior studies where TMUB1 overexpression has been correlated with malignancy in other cancer types, suggesting a general oncogenic role for TMUB1 [12]. However, the specific mechanisms through which TMUB1 promotes glioma progression warrant further investigation.

Crucially, our survival analyses underscore TMUB1's prognostic significance. Patients with high TMUB1 expression exhibited poorer overall survival, both in The Cancer Genome Atlas (TCGA) dataset and our retrospective cohort, emphasizing its potential as a prognostic indicator. These results align with findings in other cancer types, where elevated TMUB1 has been associated with adverse outcomes. The precise molecular pathways underlying TMUB1's impact on glioma patient survival remain a subject of interest for future research. For example, TUMB1 has been reported to have crosstalk with p53 signaling pathway, which is a well-recognized oncogene, in various diseases [19–21].

Furthermore, our functional experiments reveal that TMUB1 knockdown significantly inhibits glioma cell growth, offering a mechanistic understanding of its oncogenic role. This aligns with studies in other cancers where TMUB1 has been implicated in cell proliferation, apoptosis, and invasion [13, 14]. This mechanistic insight underscores the potential of TMUB1 as a therapeutic target in glioma.

While our study contributes significantly to understanding the role of TMUB1 in glioma, several limitations merit consideration. The retrospective design of this study, which exclusively enrolled patients from a single medical center in China, presents a notable limitation due to the restricted number of cases and potential biases associated with the specific geographical and demographic context. The study's exclusive focus on a particular population within a singular medical center restricts the applicability of its findings to a broader glioma patient demographic, especially those from varied geographic locations and ethnic backgrounds. The inherent diversity in glioma characteristics across different populations may influence the study's external validity, leading to conclusions that may not be universally applicable. It is essential to recognize that the demographic and clinical features of the patient cohort in this study may differ from those in other investigations conducted in diverse healthcare settings. Therefore, caution is advised when extrapolating the study's outcomes to more extensive populations, and future research endeavors with larger and more diverse cohorts are necessary to validate the identified associations. Additionally, the underlying mechanisms by which TMUB1 influences glioma progression and immune responses require further elucidation. Prospective studies and functional analyses are warranted to confirm the clinical utility of TMUB1 as a biomarker and therapeutic target. Future investigations into the molecular pathways influenced by TMUB1 and its interactions within the tumor microenvironment may offer novel therapeutic strategies for this challenging cancer.

## 5. Conclusion

In conclusion, our study unveils the multifaceted roles of TMUB1 in glioma, establishing it as a novel diagnostic biomarker and a robust prognostic indicator. The functional experiments underscore its oncogenic potential and highlight its candidacy as a therapeutic target. Correlations with immune cell infiltration suggest TMUB1's involvement in the complex immune landscape of glioma. These findings offer critical insights into the intricate biology of glioma and open doors for further research into the mechanistic underpinnings of TMUB1's functions.

## Figures and Tables

**Table 1:** Correlations between clinical characteristics and TMUB1 mRNA level in glioma patients.

Variables	Patients (*n* = 109)	TMUB1 mRNA level		*P* value
		Low (*n* = 53)	High (*n* = 56)	
Gender				0.820
Female	42	21	21	
Male	67	32	35	
Age (years)				0.630
≤50	54	25	29	
>50	55	28	27	
Tumor size				0.005^∗^
≤5.0 cm	72	42	30	
>5.0 cm	37	11	26	
WHO grade				0.001^∗^
Grade II	23	18	5	
Grade III	41	21	20	
Grade IV	45	14	31	
Karnofsky score				0.688
≤90	72	36	36	
>90	37	17	20	
Surgery				0.366
GTR	48	27	21	
STR	37	16	21	
PR/biopsy	24	10	14	

^∗^Statistically significant by chi-square test. Abbreviations: TMUB1: transmembrane and ubiquitin-like domain-containing 1; GTR: gross total resection; STR: subtotal resection; PR: partial resection.

**Table 2:** Univariate analysis for overall survival of glioma patients.

Variables	Patients (*n* = 109)	Overall survival		*P* value
		Median (months)	3-year OS	
Gender				0.208
Female	42	37.0	51.8%	
Male	67	23.0	39.1%	
Age (years)				0.292
≤50	54	22.0	47.2%	
>50	55	33.0	40.3%	
Tumor size				0.296
≤5.0 cm	72	35.0	47.4%	
>5.0 cm	37	17.0	37.2%	
WHO grade				<0.001^∗^
Grade II	23	65.0	73.9%	
Grade III	41	42.0	53.8%	
Grade IV	45	15.0	20.5%	
Karnofsky score				0.942
≤90	72	32.0	43.2%	
>90	37	34.0	45.9%	
Surgery				0.008^∗^
GTR	48	46.0	57.7%	
STR	37	22.0	40.5%	
PR/biopsy	24	17.0	19.8%	
TMUB1 mRNA level				<0.001^∗^
Low	53	48.0	66.4%	
High	56	15.0	23.1%	

^∗^Statistically significant by log-rank test. Abbreviations: GTR: gross total resection; STR: subtotal resection; PR: partial resection; TMUB1: transmembrane and ubiquitin-like domain-containing 1.

**Table 3:** Multivariate analysis for the prognostic factors of glioma patients.

Variables	Hazard ratio	95% confidence intervals	*P* value
WHO grade			
Grade II	Reference		
Grade III	1.672	0.925-3.022	0.089
Grade IV	2.995	1.609-5.575	0.001^∗^
Surgery			
GTR	Reference		
STR	1.433	0.886-2.318	0.143
PR/biopsy	2.575	1.451-4.568	0.001^∗^
TMUB1 mRNA level			
Low TMUB1 level	Reference		
High TMUB1 level	2.389	1.504-3.796	<0.001^∗^

^∗^Statistically significant by the Cox regression test. Abbreviations: HR: hazard ratio; CI: confidence intervals; TMUB1: transmembrane and ubiquitin-like domain-containing 1.

## Data Availability

Original data will be available upon rational request.
